# Supplementary material for: Towards a More Efficient In and Ex Situ Conservation of Sri Lankan Wild Rice Species
Source: Plants (Basel). 2023 May 29;12(11):2149. doi: 10.3390/plants12112149 (PMC10255471; doi:10.3390/plants12112149)
Supplement: Supplementary file 1 [file plants-12-02149-s001.zip › plants-2260648-supplementary.pdf]

Table S1. Number of seed accessions from IRRI, PGRC.

Species	IRRI	PGRC
O.nivara	29	24
O.rhizomatis	20	23
O.rufipogon	11	07
O.eichengeri	10	09
O.granulata	02	02
Total	72	65

IRRI – International Rice Research Institute, PGRC-Plant Genetic Resource Center.

Table S2. Status of *in-situ* and *ex-situ* conservation of Sri Lankan wild rice species.

Species	Number of Literature locations	Number of confirmed locations	Number of locations that fall within protected areas	Effective/ineffective *in-situ* protection	Number of *ex-situ* seed accessions	IUCN category in Sri Lanka (IUCN Sri Lanka, 2021)
O.nivara	65	13	13	Effective	29	NT
O.rhizomatis	46	26	07	ineffective	20	VU
O.rufipogan	29	8	00	ineffective	11	NT
O.eichengeri	12	3	10	ineffective	10	LC
O.granulata	14	3	02	Effective	02	NE

NT- Near Threatened, VU- Vulnerable, LC-Least Concerned, NE- Not Evaluated.

Table S3. Details of locations, reference, presence/ absence of population during field survey and presence of population in/outside the protected area of Sri Lankan wild rice species.

Species	District	Location	Latitude	Longitude	Data source	Reference	Note	Protected areas
O.nivara	Monaragala		6.4109	81.3293	Literature	Liyanage et al.,2010	-	
O.nivara	Anuradhapura		8.3108	80.3321	Literature	Liyanage et al.,2010	-	√

O.nivara	Anuradhapura		8.3768	80.0758	Literature	Liyanage et al.,2010	-	√
O.nivara	Ampara	Lahugala, Kitulana N.P – Pottuvil rd	6.8896	81.7217	Literature	Liyanage et al.,2010	-	X
O.nivara	Hambantota	Yala, Gonagala wawa, YNP	6.3349	81.4519	Literature	Liyanage et al.,2010	-	√
O.nivara	Hambantota	Gonagala wawa, YNP	6.3333	81.45	Literature	Liyanage et al.,2010	-	√
O.nivara	Hambantota	Buthawa junction, YNP	6.3184	81.4823	Literature	Liyanage et al.,2010	Field survey in 2022 - Present	√
O.nivara	Hambantota	Uraniya Wewa, YNP	6.3256	81.476	Literature	Liyanage et al.,2010	Field survey in 2022- Present	√
O.nivara	Hambantota	Buthawa,YNP	6.3274	81.4751	Literature	Liyanage et al.,2010	Field survey in 2022 - Present	√
O.nivara	Hambantota	Uththawapitiya, YNP	6.3248	81.4768	Literature	Liyanage et al.,2010	Field survey in 2022 - Present	√
O.nivara	Hambantota	Vilapalawa Wewa, YNP	6.3049	81.4416	Literature	Liyanage et al.,2010	-	√
O.nivara	Hambantota	Sithulpauwa rd, YNP	6.2964	81.4107	Literature	Liyanage et al.,2010	-	√
O.nivara	Kurunagale	Tennawewa, Katupotha, Kurunagala	7.5883	80.0583	Literature	Liyanage et al.,2010	Field survey 2021 - Extinct	X

O.nivara	Kurunagale	Yakadapotha wewa	7.7	80.1333	Literature	Liyanage et al.,2010	Field survey in 2022 - Present	X
O.nivara	Kurunagale	Avulegama, Nikawaratiya	7.68	80.2	Literature	Liyanage et al.,2010	Field survey in 2022 - Present	X
O.nivara	Anuradhapura	Thirappane	8.2269	80.5223	Literature	Liyanage et al.,2010	-	X
O.nivara	Anuradhapura	Madaradankadawala	8.55	80.85	Literature	Liyanage et al.,2010	-	X
O.nivara	Matale	Habarana	8.0133	80.7428	Literature	Liyanage et al.,2010	-	X
O.nivara	Puttalam	Madampe	7.4754	79.8293	Literature	Liyanage et al.,2010	-	X
O.nivara	Kurunagale	Wariyapola	7.6753	80.232	Literature	Liyanage et al.,2010	-	X
O.nivara	Kurunagale	Galgamuwa	7.6757	80.2321	Literature	Liyanage et al.,2010	-	X
O.nivara	Kurunagale	Thamitawewa	7.6342	80.4391	Literature	Liyanage et al.,2010		X
O.nivara	Kurunagale	Mahakeliya	7.6124	80.2714	Literature	Liyanage et al.,2010	-	X
O.nivara	Badulla	Mahiyanganaya	7.3353	80.9932	Literature	Liyanage et al.,2010	-	X
O.nivara	Monaragala	Bibila	7.1771	81.1968	Literature	Liyanage et al.,2010	Field survey in 2022- Extinct	X
O.nivara	Monaragala	Kotaweheragala	6.4666	81.4666	Literature	Liyanage et al.,2010	-	√
O.nivara	Matale	Makulugaswewa	7.8441	80.579	Literature	Liyanage et al.,2010	Field survey in 2022- Extinct	X
O.nivara	Anuradhapura	Palbadiyawa	7.8892	80.5361	Literature	Liyanage et al.,2010	-	X

O.nivara	Kurunagale	Negama	7.9688	80.3434	Literature	Liyanage et al.,2010	Field survey in 2022- Extinct	X
O.nivara	Kurunagale	Mahahttapahuwa	8.0095	80.4057	Literature	Liyanage et al.,2010	Field survey in 2022 - Present	X
O.nivara	Anuradhapura	Illukulama	8.3104	80.3323	Literature	Liyanage et al.,2010	-	X
O.nivara	Anuradhapura	Mihintale	8.3632	80.5292	Literature	Liyanage et al.,2010	-	X
O.nivara	Anuradhapura	Wagollakada	8.6612	80.8686	Literature	Liyanage et al.,2010	-	X
O.nivara	Anuradhapura	Morakewa, Maichchawa	8.4868	80.7553	Literature	Liyanage et al.,2010	-	X
O.nivara	Anuradhapura	Mankadawala, Saliyapura	8.399	80.4488	Literature	Liyanage et al.,2010	-	X
O.nivara	Anuradhapura	Mankadawala	8.4044	80.4488	Literature	Liyanage et al.,2010	Field survey in 2021 - Extinct	X
O.nivara	Anuradhapura	Punewe	8.6108	80.4709	Literature	Liyanage et al.,2010	-	X
O.nivara	Anuradhapura	Galkandegama	8.6653	80.4765	Literature	Liyanage et al.,2010	-	X
O.nivara	Polonnaruwa	Galviharaya	7.9647	81.0038	Literature	Liyanage et al.,2010	-	√
O.nivara	Matale	Sigiriya	7.9526	80.7593	Literature	Liyanage et al.,2010	Field survey in 2021 - Extinct	X
O.nivara	Matale	Sigiriya	7.9464	80.7384	Literature	Liyanage et al.,2010	-	X
O.nivara	Matale	Dambulla	7.8582	80.6593	Literature	Liyanage et al.,2010	-	X

O.nivara	Hambanto ta	Jamburagal a, YNP	6.3035	81.4254	Literatu re	Liyanage et al.,2010	-	√
O.nivara	Hambanto ta	Jamburagal a, YNP	6.3079	81.4231	Literatu re	Liyanage et al.,2010	-	√
O.nivara	Hambanto ta	Jamburagal a, YNP	6.3165	81.4256	Literatu re	Liyanage et al.,2010	-	√
O.nivara	Hambanto ta	Jamburagal a, YNP	6.3182	81.4268	Literatu re	Liyanage et al.,2010	-	√
O.nivara	Hambanto ta	Jamburagal a, YNP	6.3179	81.4263	Literatu re	Liyanage et al.,2010	-	√
O.nivara	Hambanto ta	Unawa, YNP	6.3299	81.44	Literatu re	Liyanage et al.,2010	-	√
O.nivara	Hambanto ta	Mahasilaw a wewa, YNP	6.2919	81.4339	Literatu re	Liyanage et al.,2010	-	√
O.nivara	Hambanto ta	Uraniya rd, YNP	6.308	81.448	Literatu re	Liyanage et al.,2010	-	√
O.nivara	Hambanto ta	Palugaswal a, YNP	6.3182	81.477	Literatu re	Liyanage et al.,2010	-	√
O.nivara	Hambanto ta	Near Karuwalag aswala, YNP	6.3249	81.4728	Literatu re	Liyanage et al.,2010	-	√
O.nivara	Hambanto ta	Butthawala , YNP	6.3324	81.4743	Literatu re	Liyanage et al.,2010	-	√
O.nivara	Hambanto ta	Rakinawal a, YNP	6.3602	81.5066	Literatu re	Liyanage et al.,2010	-	√
O.nivara	Hambanto ta	Hadunoru wa wewa, YNP	6.1666	80.9116	Literatu re	Liyanage et al.,2010	Field survey in 2022- Extinct	√
O.nivara	Hambanto ta	Suduwelim ulla, YNP	6.3631	81.5138	Literatu re	Liyanage et al.,2010	-	√
O.nivara	Anuradha pura	Habarana	8.0559	80.722	Literatu re	Liyanage et al.,2010	-	X

O.nivara	Anuradha pura	Habarana	8.089	80.6627	Literatu re	Liyanage et al.,2010	-	X
O.nivara	Anuradha pura	Thirappane	8.2458	80.5215	Literatu re	Liyanage et al.,2010	-	X
O.nivara	Monaraga la	Kotiyagala	6.7795	81.5377	Literatu re	Liyanage et al.,2010	-	√
O.nivara	Hambanto ta	Patalangala	6.3581	81.504	Literatu re	Liyanage et al.,2010	Field survey in 2022 - Present	√
O.nivara	Badulla	Thalguspiti ya	7.29	81.0208	Literatu re	Liyanage et al.,2010	-	X
O.nivara	Hambanto ta	Gonagalapi tiya	6.3468	81.4453	Literatu re	Liyanage et al.,2010	-	√
O.nivara	Badulla	Udaya Raja Mawatha	6.9861	81.0576 6	Literatu re	Liyanage et al.,2010	Field surey in 2022 - Extinct	
O.nivara	Kurunagal e	Kolamunna wewa	7.5618	80.1902	Literatu re	Liyanage et al.,2010	-	X
O.nivara	Kurunagal e	Dematawe wa	7.9869	80.3165	Literatu re	Liyanage et al.,2010	-	X
O.nivara	Anuradha pura	Mihintale	8.3546	80.4712	Literatu re	Liyanage et al.,2010	-	X
O.nivara	Anuradha pura	Mawathaw ewa	8.2833	80.2	Literatu re	Liyanage et al.,2010	-	X
O.nivara	Anuradha pura	Ambagusw ewa	8.6073	80.8391	Literatu re	Liyanage et al.,2010	-	X
O.nivara	Anuradha pura	Negama	7.9823	80.4779	Literatu re	Liyanage et al.,2010	-	X
O.nivara	Jaffna	Chavakach cheri	9.672722	80.1651 9	Literatu re		Field survey in 2021 – New population	X
O.nivara	Jaffna	Thanankila ppu	9.63425	80.1490 8333	Literatu re	-	Field survey in 2021 –	X

							New population	
O.nivara	Jaffna	Manduvil	9.675222	80.17836	Literature	-	Field survey in 2021 – New population	X
O.nivara	Jaffna	Kalvayal	9.668995	80.162466	Literature	-	Field survey in 2021 – New population	X
O.nivara	Jaffna	Kopay South	9.69885	80.063319	Literature	-	Field survey in 2021 – New population	X
O.rhizomatis	Puttalam	Eluwankulam	8.086389	79.84528	Literature	Liyanage et al.,2010	Field survey in 2021 – Present	X
O.rhizomatis	Puttalam	Madurankuliya	7.876111	79.81583	Literature	Liyanage et al.,2010	Field survey in 2021 – Present	X
O.rhizomatis	Puttalam	Welpittaniya	8.266389	79.89056	Literature	Liyanage et al.,2010	Field survey in 2021 – Present	X
O.rhizomatis	Puttalam	Tabbowa	8.055278	79.927500	Literature	Liyanage et al.,2010	Field survey in 2021 – Present	X
O.rhizomatis	Puttalam	Sella Kandal	8.044111	79.918	Literature	Liyanage et al.,2010	Field survey in 2021 – Present	X
O.rhizomatis	Puttalam	Sella kandal	8.046694	79.926	Literature	Liyanage et al.,2010	Field survey in 2021 – Present	X
O.rhizomatis	Puttalam	Vattakandal	8.118194	79.856	Literature	Liyanage et al.,2010	Field survey in 2021 – Present	X

O.rhizomatis	Puttalam	Kaladiya	7.982889	79.907	Literatu re	Liyanage et al.,2010	Field survey in 2021 – Present	X
O.rhizomatis	Anuradha pura	Oyamadu wa	8.442306	80.279	Literatu re	Liyanage et al.,2010	Field survey in 2021 – Present	X
O.rhizomatis	Anuradha pura	Medinnoru wa, Meegalewa	8.054111	80.364	Literatu re	Liyanage et al.,2010	Field survey in 2021 – Present	X
O.rhizomatis	Monaraga le	Sella Kataragam a	6.461611	81.3108 1	Literatu re	Liyanage et al.,2010	Field survey in 2021 – Present	√
O.rhizomatis	Monaraga le	Sella Kataragam a	6.443194	81.3098 1	Literatu re	Liyanage et al.,2010	Field survey in 2021 – Present	X
O.rhizomatis	Monaraga le	Sella Kataragam a	6.411	81.3436 9	Literatu re	Liyanage et al.,2010	Field survey in 2021 – Present	√
O.rhizomatis	Monaraga le	Sella Kataragam a	6.578	81.2798 1	Literatu re	Liyanage et al.,2010	Field survey in 2021 – Present	√
O.rhizomatis	Hambanto ta	Andaragas yaya	6.22	81.31	Literatu re	Liyanage et al.,2010	Field survey in 2021 – Present	√
O.rhizomatis	Hambanto ta	Kirinda	6.3585	81.5	Literatu re	Liyanage et al.,2010	Field survey in 2021 – Present	√
O.rhizomatis	Hambanto ta	Manic ganga, YNP	6.3708	81.5186	Literatu re	Liyanage et al.,2010	Field survey in 2021 – Present	√
O.rhizomatis	Hambanto ta	Yala wewa, YNP	6.3637	81.5141	Literatu re	Liyanage et al.,2010	Field survey in 2021 – Present	√
O.rhizomatis	Puttalam	Karikattiya	7.948806	79.828	literatur e	Liyanage A.S.U and Senanayak e G., 2017	-	X

O.rhizomatis	Puttalam	Nikaweratiya	7.703889	80.16417	literature	Ratnayake et al.,2021	-	X
O.rhizomatis	Puttalam	Pawattamaduwa	8.073806	79.91069	literature	Ratnayake et al.,2021	-	X
O.rhizomatis	Anuradhapura	Ganewalpola	8.095	80.61111	literature	Liyanage et al., 2002	-	X
O.rhizomatis	Anuradhapura	Maha Bulankulama	8.305556	80.31889	literature	Liyanage et al., 2002	-	X
O.rhizomatis	Anuradhapura	Rajanganaya	8.227306	80.0885	literature	Liyanage A.S.U and Senanayake G., 2017	Field survey in 2021 – Extinct	X
O.rhizomatis	Anuradhapura	Nochchiyagama	8.326306	80.13761	literature	Ratnayake et al.,2021	Field survey in 2021 – Present	X
O.rhizomatis	Anuradhapura	Sangilikanadarawa Wewa	8.527306	80.52731	literature	Ratnayake et al.,2021	Field survey in 2021 – Extinct	X
O.rhizomatis	Anuradhapura	Andarawewa	8.279306	80.26431	literature	Ratnayake et al.,2021	Field survey in 2021 – Present	X
O.rhizomatis	Kurunagale	Nikaweratiya	7.703889	80.16417	Literature	Liyanage et al., 2002	Field survey in 2021 – Extinct	X
O.rhizomatis	Kurunagale	Medinnoruwa, Meegalewa	8.054111	80.364	Literature	Ratnayake et al.,2021	Field survey in 2021 – Present	X
O.rhizomatis	Kurunagale	Walaswewa	8.030611	80.319	Literature	Ratnayake et al.,2021	Field survey in 2021 – Extinct	X
O.rhizomatis	Ampara	Perani Lahugala	6.888694	81.6945	literature	Ratnayake et al.,2021	-	X
O.rhizomatis	Hambantota	Colombo - Galle - Hambantota -	6.483	81.13081	Literature	Ratnayake et al.,2021	-	X

		Wellawaya Rd						
0. eichingeri	Matale	Archeoogical site, Manikdena, Lenadora, Dambulla	7.7646	80.6399	Literature	Liyanage et al.,2010		X
0. eichingeri	Ratnapura	Water canal, before Waulpane school, Waulpane, Ratnapura	6.4317	80.7281	Literature	Liyanage et al.,2010	Field survey in 2021 – Present	X
0. eichingeri	Ratnapura	Waulpane, Ratnapura	6.4281	80.7309	Literature	Liyanage et al.,2010	Field survey in 2021 – Present	X
O.eichingeri	Matale	Watapitiya, Wasgamuwa National park	7.6636	80.9989	Literature	Liyanage et al.,2010	-	X
O.eichengeri	Matale	Karpinchaela, Wasgamuwa National ark	7.6636	80.9989	Literature	Liyanage et al.,2010	-	X
O.eichengeri	Matale	Medapitiya, wasgamuwa National park	7.6716	80.9271	Literature	Liyanage et al.,2010	-	√
O.eichengeri	Matale	Mukkurakanda, Wasgamuwa National park	7.6835	80.9009	Literature	Liyanage et al.,2010	-	√

O.eichengeri	Matale	Ulpath Hatha camp site, Wasgamu wa National Park	7.6816	80.8975	Literatu re	Liyanage et al.,2010	-	√
O.eichengeri	Kandy	Telwela, Heen Ganga Anicut, Road to Dungolla	7.4629	80.9469	Literatu re	Liyanage et al.,2010	-	X
O.eichengeri	Polonnaru wa	Unapitiya, Minneriya National Park, Polonnaru wa	8.0222	80.8384	Literatu re	Liyanage et al.,2010	-	X
O.eichengeri	Polannaru wa	Akkara 100 para, Minneriya National park, Polannaru wa	8.0323	80.8335	Literatu re	Liyanage et al.,2010		X
O.eichengeri	Ratnapura	Rajawaka, Balangoda Thanamali wila rd	6.6079	80.7932	Literatu re	Liyanage et al.,2010	Field survey in 2021 - Present	X
O.eichengeri	Anuradha pura	Bonji temple,Mih intale Mahiyanga na	8.3459	80.5058	Literatu re	Liyanage et al.,2010	-	X
O.eichengeri	Matale	Thelwala, Heen ganga,	7.9257	80.9977	Literatu re	Liyanage et al.,2010	-	X

		wilgamuwa						
O.eichengeri	Anuradhapura	Ritigala	8.1176	80.5813	Literature	Liyanage et al.,2010	-	X
O.eichengeri	Matale	Archeoogical site, Manikdena, Lenadora, Dambulla	7.7674	80.6074	Literature	Liyanage et al.,2010	-	X
O.eichengeri	Polonnaruwa	Hatharas kotuwa, Galoyahandiya	8.1464	80.8449	Literature	Liyanage et al.,2010	-	X
O.eichengeri	Monaragala	Makara rd, Galoya National Park	7.1911	81.4223	Literature	Liyanage et al.,2010	-	√
O.eichengeri	Monaragala	Seenukkanda, Galoya national Park	7.1885	81.3784	Literature	Liyanage et al.,2010	-	√
O.granulata	Monaragale	Wellawaya	6.7281	81.084	Literature	Liyanage et al.,2010	-	X
O.granulata	Ratnapura	Kapugala	6.5825	80.7989	Literature	Liyanage et al.,2010	Field survey in 2021 - Present	X
O.granulata	Matale	Sigiriya	7.9505	80.7744	Literature	Liyanage et al.,2010	-	X
O.granulata	Matale	Sigiriya	7.9498	80.779	Literature	Liyanage et al.,2010	-	X
O.granulata	Ratnapura	Waulpane	6.4215	80.733	Literature	Liyanage et al.,2010	Field survey in 2021 - Present	X
O.granulata	Ratnapura	Waulpane	6.4333	80.7333	Literature	Liyanage et al.,2010	Field survey in 2021 - Present	X

O.granulata	Ratnapura	Waulpane	6.4166	80.7166	Literature	Liyanage et al.,2010	Field survey in 2021 - Present	X
O.granulata	Monaragala	Wellawaya	6.7281	81.0849	Literature	Liyanage et al.,2010	-	X
O.granulata	Badulla	Beragala	6.716	81.0669	Literature	Liyanage et al.,2010	-	X
O.granulata	Ratnapura	Daminakatiya	6.4015	80.7216	Literature	Liyanage et al.,2010	-	X
O.granulata	Ratnapura	Ellepahala	6.4019	80.7216	Literature	Liyanage et al.,2010	-	X
O.granulata	Monaragala	Bergala	6.7333	81.1	Literature	Liyanage et al.,2010	-	X
O.rufipogon	Kalutara	Bandaragama	6.7095	79.97875	Literature	Liyanage et al.,2010	Field survey in 2021 - Present	X
O.rufipogon	Kalutara	Moronthuduwa	6.6355833	79.9509166	Literature	Liyanage et al.,2010	Field survey in 2021 - Present	X
O.rufipogon	Kalutara	Wadduwa	6.6665833	79.960611	Literature	Liyanage et al.,2010	Field survey in 2021 - Present	X
O.rufipogon	Kalutara	Palpola	6.6359167	80.01066	Literature	Liyanage et al.,2010	Field survey in 2021 - Present	X
O.rufipogon	Kalutara	Delkada Pelpola	6.64644	79.99316	Literature	Liyanage et al.,2010	Field survey in 2021 - Present	X
O.rufipogon	Kalutara	Morontuduwa	6.669916	79.9812	Literature	Liyanage et al.,2010	Field survey in 2021 - Present	X
O.rufipogon	Kalutara	Thoragala	6.6290833	79.987	Literature	Liyanage et al.,2010	Field survey in 2021 - Present	X

O.rufipogon	Matara	Palatuwa	5.991916 7	80.5199 16	Literatu re	Liyanage et al.,2010	Field survey in 2021 - Present	X
O.rufipogon	Hambanto ta	Weeraketiy a	6.155166 7	80.7681	Literatu re	Liyanage et al.,2010	-	X
O.rufipogon	Kalutara	Paraduwa	6.627777	79.9875	Literatu re	-	Confirmed in 2021	X
O.rufipogon	Kalutara	Panapitiya South	6.6277	79.9575	Literatu re	-	Confirmed in 2021	X

Table S4. Ex situ seed bank data from IRRI.

Species	Seed bank	Collection site	Accessio n number	Acq-date	Latitude	Longitude
0. eichingeri	IRRI	Katharagama	81803	2/28/1992	6.416667	81.3331
0. eichingeri	IRRI	Nindapella	81804	2/28/1992	7.611667	80.47222
0. eichingeri	IRRI	Dambulla	101442	4/27/1964	7.876944	80.7
0. eichingeri	IRRI	Godakawela	104608	3/5/1984	6.5	80.6667
0. eichingeri	IRRI	Pallegama	105407	2/14/1988	7.5	80.6667
0. eichingeri	IRRI	Pallegama	105408	2/14/1988	7.5	80.6667
0. eichingeri	IRRI	Mahasengama	105412	2/14/1988	8	80.8333
0. eichingeri	IRRI	Unagollewa Wewa	105413	2/14/1988	8.166667	80.6667
0. eichingeri	IRRI	Unagollewa Wewa	105414	2/14/1988	8.166667	80.6667

0. eichingeri	IRRI	Unagollewa Wewa	105415	2/14/1988	8.166667	80.6667
O.granulata	IRRI	Bandaragama	100880	1/27/1963	6.716667	79.9833
O.granulata	IRRI	Dambulla	104611	3/5/1984	8	80.6667
O. nivara	IRRI	Thihilivaddai	101994	6/8/1972	7.833333	81.5208
O. nivara	IRRI	Dambulla	103407	2/1/1978	7.876944	80.7
O. nivara	IRRI	Paranahalmillew a	103415	2/26/1979	8.65	80.5
O. nivara	IRRI	Alagalla	103416	2/26/1979	8.666667	80.5
O. nivara	IRRI	Vavuniya	103418	2/26/1979	8.666667	80.4167
O. nivara	IRRI	-	103419	2/26/1979	9.666667	80.4167
O. nivara	IRRI	Odavi Kulam	103420	2/26/1979	8.916667	81
O. nivara	IRRI	Odavi Kulam	103422	2/26/1979	8.916667	81
O. nivara	IRRI	Dunumadalawa	104612	3/5/1984	7.946944	80.6258
O. nivara	IRRI	Mahasengama	105409	2/14/1988	8	80.8333
O. nivara	IRRI	Mahasengama	105410	2/14/1988	8	80.8333
O. nivara	IRRI	Tammannawa Wewa	105416	2/14/1988	8.083333	80.6667
O. nivara	IRRI	Tammannawa Wewa	105417	2/14/1988	8.083333	80.6667
O. nivara	IRRI	Vihara Palugama	105418	2/14/1988	8.333333	80.3333
O. nivara	IRRI	Vihara Palugama	105419	2/14/1988	8.333333	80.3333
O. nivara	IRRI	-	105428	2/14/1988	6.166667	81.5
O. nivara	IRRI	-	105430	2/14/1988	6.166667	81.5
O. nivara	IRRI	-	105431	2/14/1988	6.166667	81.5
O. nivara	IRRI	-	105433	2/14/1988	6.166667	81.5
O. nivara	IRRI	-	105434	2/14/1988	6.166667	81.5
O. nivara	IRRI	-	105442	2/14/1988	6.166667	81.5

O. nivara	IRRI	-	105444	2/14/1988	6.166667	81.5
O. nivara	IRRI	Thampalawela	105453	2/14/1988	7	81.3333
O. nivara	IRRI	Kandakettiya Town	105454	2/14/1988	7.166667	81
O. nivara	IRRI	Kandakettiya Town	105455	2/14/1988	7.166667	81
O. nivara	IRRI	Kandakettiya Town	105456	2/14/1988	7.166667	81
O. nivara	IRRI	Kandakettiya Town	105458	2/14/1988	7.166667	81
O. nivara	IRRI	Puliyan Kulam	105459	2/14/1988	8	81.1667
O. nivara	IRRI	Kandalama Reservoir	106188		7.876944	80.7
O. rhizomatis	IRRI	Nindapella	86466	2/28/1992	7.611667	80.4722
O. rhizomatis	IRRI	Murukkuwataw ana	103410	2/26/1979	8.083333	80
O. rhizomatis	IRRI	Thamarakkulam a	103414	2/26/1979	7.75	79.9167
O. rhizomatis	IRRI	Echchankulam	103417	2/26/1979	8.833333	80.4167
O. rhizomatis	IRRI	Murukkuwataw ana	103421	2/26/1979	8.916667	81
O. rhizomatis	IRRI	Elalla	104609	3/5/1984	6.333333	81.0833
O. rhizomatis	IRRI	-	105429	2/14/1988	6.166667	81.5
O. rhizomatis	IRRI	-	105432	2/14/1988	6.166667	81.5
O. rhizomatis	IRRI	-	105440	2/14/1988	6.166667	81.5
O. rhizomatis	IRRI	-	105443	2/14/1988	6.166667	81.5
O. rhizomatis	IRRI	-	105445	2/14/1988	6.166667	81.5
O. rhizomatis	IRRI	-	105446	2/14/1988	6.166667	81.5
O. rhizomatis	IRRI	Patanagala Point	105447	2/14/1988	6.333333	81.5
O. rhizomatis	IRRI	Patanagala Point	105448	2/14/1988	6.333333	81.5
O. rhizomatis	IRRI	Kirinda	105449	2/14/1988	6.5	81.5

O. rhizomatis	IRRI	Kirinda	105450	2/14/1988	6.5	81.5
O. rhizomatis	IRRI	Katupathwewa	105659	2/14/1988	8.25	80.0833
O. rhizomatis	IRRI	-	105660	2/14/1988	6.166667	81.5
O. rhizomatis	IRRI	-	105949	2/14/1988	6.166667	81.5
O. rhizomatis	IRRI	-	105950	2/14/1988	6.166667	81.5
O. rufipogon	IRRI	Kandalama Reservoir	99526	2/14/1993	7.876944	80.7
O. rufipogon	IRRI	Nabadawewa	104599	3/5/1984	7.666667	80.1667
O. rufipogon	IRRI	-	104602	3/5/1984	7.5	79.8333
O. rufipogon	IRRI	Wendesiwatta 1st Ln, Wattala	104605	3/5/1984	7	79.9167
O. rufipogon	IRRI	Paragasthota	105420	2/14/1988	6.666667	80
O. rufipogon	IRRI	Paragasthota	105422	2/14/1988	6.666667	80
O. rufipogon	IRRI	Munhena	105424	2/14/1988	6.5	80
O. rufipogon	IRRI	Akurugoda South	105425	2/14/1988	6	80.5
O. rufipogon	IRRI	Akurugoda South	105426	2/14/1988	6	80.5
O. rufipogon	IRRI	-	105461	2/14/1988	7	80
O. rufipogon	IRRI	-	105463	2/14/1988	7	80
